# Absence of Adolescent Obesity in Grenada: Is This a Generational Effect?

**DOI:** 10.3389/fpubh.2018.00204

**Published:** 2018-08-03

**Authors:** Richard A. Scribner, Roger L. Radix, Aubrey E. Gilliland, Claudia Leonardi, Tekeda F. Ferguson, Trevor P. Noel, Rebecca G. Andall, Naomi R. Andall, Christal Radix, Rhoda Frank, Jonell Benjamin, Jenifer James, Romero Benjamin, Randall L. Waechter, Melinda S. Sothern

**Affiliations:** ^1^Epidemiology Department, Louisiana State University Health Sciences Center School of Public Health, New Orleans, LA, United States; ^2^Stanley S. Scott Cancer Center, Louisiana State University Health Sciences Center School of Medicine, New Orleans, LA, United States; ^3^Windward Islands Research and Education Foundation, St. George's University, St. George's, Grenada; ^4^Behavioral and Community Health Sciences Department, Louisiana State University Health Sciences Center School of Public Health, New Orleans, LA, United States; ^5^Department of Pediatrics, Louisiana State University Health Sciences Center School of Public Health, New Orleans, LA, United States

**Keywords:** obesity, body mass index, adolescent, generation, nutrition, cohort effect, diet quality

## Abstract

**Background:** Low- and middle-income countries are affected disproportionately by the ongoing global obesity pandemic. Representing a middle income country, the high prevalence of obesity among Grenadian adults as compared to US adults is expected as part of global obesity trends. The objective of this study was to determine if Grenadian adolescents have a higher prevalence of overweight compared to their US counterparts, and if a disparity exists between urban and rural adolescents.

**Methods:** Using a subcohort of participants in the Grenadian Nutrition Student Survey, diet quality and anthropometric measures were collected from 55% of the classrooms of first year secondary students in Grenada (*n* = 639). Rural or urban designations were given to each school. Body Mass Index (BMI) was calculated and categorized as overweight or obese for each student following CDC classification cutoffs. A standardized BMI (BMIz) was calculated for each school. Sex-specific BMI and overall BMIz were compared to a 1980s US cohort. Multilevel models, overall and stratified by sex, of students nested within schools were conducted to determine if BMIz differed by rural or urban locality, gender, and diet quality.

**Results:** The mean age of this cohort was 12.7 (*SD* = 0.8) years with 83.8% of the cohort identifying as Afro-Caribbean. Females had nearly twice the prevalence of overweight when compared to males (22.7 vs. 12.2%) but a similar prevalence of obesity (8.2 vs. 6.8%). Grenadian adolescents had lower prevalence of overweight (females: 22.7 vs. 44.7%; males: 12.2 vs. 38.8%, respectively) as compared to US counterparts. Eating a traditional diet was negatively associated with BMIz score among females ($\hat{\beta }$ = −0.395; SE = 0.123) in a stratified, multilevel analysis. BMIz scores did not differ significantly by rural or urban school designation.

**Conclusions:** Among Grenadian adolescents, this study identified a lower overweight prevalence compared to US counterparts and no difference in overweight prevalence by urban or rural location. We hypothesize that the late introduction of processed foods to Grenada protected this cohort from obesogenic promoters due to a lack of fetal overnutrition. However, further research in subsequent birth cohorts is needed to determine if adolescent obesity will increase due to a generational effect.

## Introduction

The epidemiologic transition from communicable disease and nutritional deficiency to non-communicable disease is occurring in low- and middle-income countries (LMIC) (1). It is widely recognized that this transition is primarily the result of the global obesity pandemic leading to increased rates of cardiovascular disease, diabetes and cancer (1, 2). Developed countries underwent the epidemiologic transition decades ago, and indications of the forthcoming obesity pandemic were seen as early as the 1980s (3). Since then, obesity prevalence has doubled among adults and tripled among children in the United States (US) (4). These increases of obesity highlight the dietary and social changes the US and other developed countries underwent in the last half of the 20th century. Furthermore the obesity pandemic is widely attributed to a global infrastructure that makes highly processed foods widely available in terms of cost and access (5). The proliferation of industrialized food processing, as well as advances in technology that allow for decreased physical activity, have largely been held as the main reasons behind the pandemic (6–9). These obesogenic lifestyle changes have been proliferating globally since normalizing in developed countries, and the health effects are just now being realized in LMIC (3, 5).

In an effort to understand societal exposures driving the global pandemic, Monteiro and Cannon (10) suggest the widespread availability of ultra-processed foods was temporally associated with the obesity epidemic in Brazil. They demonstrated the dramatic rise in obesity in Brazil during the 1990s was not associated with any particular nutrient (e.g., sugar, fat) but with replacement of minimally processed, traditionally prepared foods with the ultra-processed foods produced by transnational food companies (11, 12). The rapid globalization of the corporate food infrastructure over the past decades has made the introduction of ultra-processed foods occur more rapidly in LMIC compared to what was seen with their introduction in high income countries in earlier decades (12). From 1998 to 2012, LMIC more than doubled their spending on processed foods and quadrupled spending on soft drinks. Relative growth of sales of these processed foods had an inverse relationship with gross national income, indicating LMIC increase spending on these products annually (13). Growth in processed food sales have positive implications for the prevalence of underweight, which is declining, but conversely negative implications for prevalence of overweight, which continues to increase worldwide (13). This may account for the rapid transitions now occurring in many LMIC countries.

Studies reviewing childhood obesity trends among LMIC in the Caribbean are inconsistent. The prevalence of overweight/obese (OW/OB) increased from 8.52% in 1981 to 32.5% in 2010 among 9–11 year olds in Barbados, and a slightly older age group (14–19 year olds) in Jamaica had a similar prevalence of OW/OB of 35.5% in 2007 (14, 15). Conversely, in a cohort of 7–18 year olds in Trinidad, only 17% were OW/OB in 2010 (16). These discrepancies in childhood obesity prevalence in the Caribbean may be explained by differences in country income. Out of these three countries, Trinidad has experienced the greatest growth in income over the past few decades, almost double that of Jamaica and five times that of Barbados (17). After reviewing the findings of Monteiro and colleagues mentioned previously, Trinidad may have concurrently experienced decreases or stagnation in spending on processed foods, explaining why childhood obesity prevalence is lowest in this country among the three (13).

The built environment is also an important factor to assess when measuring obesity. In high-income countries, the prevalence of obesity is higher in rural areas as compared to urban (18). With the development of LMIC resulting in the introduction of ultra-processed foods, investigators are seeing the burden of obesity shifting to or equalizing with urban areas (19). A recent study determined that Nicaraguan urban overweight prevalence experienced greater increases than rural, with an annualized change in the prevalence of overweight in the 1990s−2000s of 2.33 vs. 1.00, respectively (20). Yet, in other countries, such as Haiti, increases in obesity prevalence have stabilized between urban and rural environments (0.73 vs. 0.73) (20). This inconclusive information regarding the obesity prevalence disparity between urban and rural environments within LMIC warrants further investigation.

Since 1990, Latin America and the Caribbean have experienced an average increase of 0.31% in OW/OB prevalence annually (20). Grenada represents a LMIC currently undergoing the epidemiologic transition, of which increases in OW/OB prevalence are a direct side effect (21). However, there have been inconsistent findings regarding obesity prevalence among the Grenada population. A recent study indicates the prevalence of overweight and obesity (OW/OB) among adult Grenadians is 57%, which is comparable to US black adults (22). In contrast, a report conducted by the Caribbean Food and Nutrition Institute demonstrated low rates of OW/OB among a sample of Grenadian pre-school children compared to US counterparts (23). Research to determine if Grenadian adolescent obesity has a higher prevalence of OW/OB as compared to a similar US cohort is lacking.

Given these contradictory reports, the objective of the present study was to determine if Grenadian adolescents have a higher prevalence of OW/OB compared to their US counterparts, and if a disparity exists between urban and rural adolescents.

## Materials and methods

### Study participants

In an effort to develop policies to prevent obesity among youth, the Windward Islands Research and Education Foundation (WINDREF) at St. George's University (SGU) conducted a nutrition survey of Grenadian adolescents called the Grenada School Nutrition Study (GSNS). The current study cohort is a subset of adolescents who participated in the GSNS. Institutional review boards at both SGU and Louisiana State University Health Sciences Center approved the protocols. At least one classroom of first year secondary students (i.e., Form 1) was sampled from each of the 23 Grenadian secondary schools. Although the target age range was 11–14 years old, all students within a given class were eligible to participate regardless of age. During the 2012–2013 academic year, 1,004 students were sampled from 35 classrooms, constituting approximately 55% of all Form 1 students in Grenada. Parental refusals (*n* = 276) and students who were absent or did not complete all the assessments or surveys (*n* = 39) resulted in a final study cohort of 689 students.

### Data collection

Students from two schools were recruited each week to participate in a demographic survey, an anthropometric assessment, and a food frequency questionnaire specific to the Grenadian diet, adapted from the Eating Habits Questionnaire (EHQ) (24). Assessments and surveys were administered on two successive days in a classroom, activity room, or gym that was allocated by each school for the project. The surveys were interviewer assisted. A team of interventionists implemented the procedures simultaneously. Each interventionist was certified by GSNS co-investigators to ensure performance standards were met. All procedures, testing methods and measurement tools were pilot tested and revised as necessary prior to final data collection. On day 1, anthropometric data (e.g., height, weight, waist circumference) were collected, and nutrition information, such as portion size, was provided to the student, which was necessary to ensure accurate completion of the food frequency questionnaire. On day 2, the demographic survey (including medical/family history) and food frequency questionnaire were administered. Scheduling of data collection days avoided dates subject to potential seasonal biases related to holidays, exams, and school events. Rural and urban designations were defined at the school level, with students attending school in the two largest towns in Grenada (St. Georges and Grenville) defined as urban students.

### Anthropometric measures

Height, weight, and waist circumference were directly measured for each student in a private setting (e.g., trifold blind booth). The students wore school uniforms and were asked to remove all outer clothing, heavy pocket items, and shoes. Height was measured twice using a portable stadiometer (Shorr Productions, Olney, Maryland) and weight was measured three times using an electronic scale (Seca 880, Seca Corporation, Hanover, Maryland), with both devices standardized before each session. The average of both measurements was used for analysis. The reliability of these measurements were established previously (25). Body Mass Index (BMI), calculated by dividing participants' weight in kilograms by height in meters squared, was used to determine excess body weight. The CDC age- and sex-specific cutoffs were used to characterize overweight (OW, BMI ≥ 85th percentile) and obese (OB, BMI ≥ 95th percentile) adolescents in terms of their standardized percentile (26). Furthermore, a standardized BMI (BMIz) for each school was calculated using the above mentioned classification system. CDC classification cutoffs are based on growth charts derived from National Health and Nutrition Examination Survey (NHANES) II (1976–1980) and NHANES III (1988–1994) (27). By using these CDC classification cutoffs, we are considering this cohort (NHANES 1976–1980, NHANES III 1988–1994) as our comparison US adolescent cohort.

### Diet quality measures

Diet quality indexes were derived from the food frequency questionnaire administered to all students. Several indexes of diet quality were developed including: the Traditional Prepared Food Index (TPFI), the Ultra Processed Food Index (UPFI), and the Sugar Sweetened Beverage Index (SSBI). The food frequency questionnaire was adapted from a previously validated food frequency questionnaire (EHQ) for use in a West Indian population to make it culturally appropriate in terms of inclusion of local dietary items (e.g., callaloo soup), use of proper dietary terms for local foods, and food models depicting typical foods and serving sizes (24). The TPFI was an index of the average number of times per week (i.e., 1 = never eaten; 2 = not eaten last week; 3 = 1–2 times per week; 4 = 3–6 times per week and 5 = almost every day) students stated they had eaten the following seven common traditional prepared foods including: porridge, oil down, pelau, fish broth, cou cou, callaloo, and provisions. The UPFI was an index of the average number of times per week students stated they had eaten the following six common ultra-processed foods including: lunch meat, hot dogs, ice cream, candy bars, presweetened cereal, and potato chips. The UPFI was adapted from processed food classifications defined by Monteiro et al. (13). The SSBI was an index of the average number of times per week students stated they drank regular soda and/or fruit flavored packaged drinks.

### Statistical analysis

All analyses were conducted using SAS statistical software package version 9.4. Summary statistics were produced using either the MEANS or FREQ procedures. Demographic characteristics, diet quality measures, and anthropometrics measures were conducted for the overall study population and also stratified by gender. Differences between genders were established using Wald tests from the MIXED procedure for continuous variables and Chi-Square Tests for Independence for categorical variables. Multilevel models of students nested within schools were developed to characterize the overall and between school BMIz across Grenada. Data were analyzed implementing random intercept models using the MIXED procedure. The outcome modeled was BMIz as a continuous variable. An empty model and various combinations of individual and multilevel measures were analyzed. Individual level variables consisted of age, gender, race, and various indices of diet quality (i.e., TPFI, UPFI, and SSBI). The only multilevel variable considered was urban/rural designation. Another multilevel analysis adjusting for variables found to be significant in the saturated multilevel model was stratified by gender. When a significant interaction between a categorical and quantitative variable was observed, a secondary analysis was conducted to investigate the estimated effect of the quantitative variable within each level of the categorical variable. Significance was defined at *p* < 0.05.

## Results

### Study population demographics

The average age of the students was 12.7 years (standard deviation (SD) = 0.8) (Table 1). Male students, on average, were significantly older than female (12.8 vs. 12.5 years old, *p* < 0.0001). In terms of ethnicity, 83.3% of the students identified as Afro Caribbean. Female students were more likely to attend rural schools. There were no statistically significant differences between males and females in terms of the diet quality items.

**Table 1:** Descriptive statistics for Grenadian adolescent participants overall and by gender.

	**Overall**		**Male**		**Female**		
	***N***	**Mean (*SD*)**	***N***	**Mean (*SD*)**	***N***	**Mean (*SD*)**	***p*-value**
Age, years	689	12.7 (0.8)	337	12.8 (0.9)	352	12.5 (0.6)	< 0.0001
TPFI^a^	628	2.4 (0.6)	296	2.5 (0.6)	332	2.4 (0.5)	0.479
UPFI^b^	635	2.7 (0.7)	303	2.7 (0.7)	332	2.7 (0.7)	0.4052
SSBI^c^	563	2.5 (1.0)	261	2.5 (1.0)	302	2.5 (1.0)	0.4901
Afro Caribbean, %	689	83.3	337	86.1	352	80.7	0.0587
Male, %	689	48.9	–	–	–	–	–
Rural, %	689	61	337	56.4	352	65.3	0.0159

a *TPFI, Traditionally Prepared Food Index*.

b *UPFI, Ultra Processed Food Index*.

c *SSBI, Sugar Sweetened Beverage Index*.

### Overweight/obese prevalence

The overall prevalence of OW/OB among Form 1 Grenadian adolescents was 17.6 and 7.6%, respectively (Table 2). Females were at a statistically significant greater risk of being OW than males (i.e., 22.7 vs. 12.2%), indicating a gender difference in the prevalence of OW/OB among Grenadian students. OB prevalence and waist circumference were not significantly different between male and female students. Grenadian female and male adolescents are less OW/OB than their US counterparts, with American girls experiencing an OW/OB prevalence of 44.7% vs. Grenadian girls at 22.7% and American boys experiencing an OW/OB prevalence of 38.8% vs. Grenadian boys at 12.2% (27). In addition, Grenadian adolescents had a significantly lower mean BMIz (−0.097; standard error (*SE*) = 0.045) compared with the US median BMI established using NHANES data from the 1980s (27).

**Table 2:** Mean (± SE) and prevalence of anthropometric measures for Grenadian adolescents overall and by gender.

	***N***	**Overall**	**Male**	**Female**	***p*-value**
Waist circumference, cm	688	65.0 (0.3)	64.5 (0.5)	65.4 (0.5)	0.159
BMIz	689	−0.097 (0.045)	−0.292 (0.063)	0.090 (0.062)	< 0.0001
BMI > 85th, % OW^a^	689	17.6	12.2	22.7	0.0003
BMI > 95th, % OB^b^	689	7.6	6.8	8.2	0.4825

a *OW, overweight*.

b *OB, obese*.

### Between school BMIz

An empty multilevel model (Table 3) with obesity risk as the outcome yielded an intraclass correlation coefficient of 0.051 indicating that 5.1% of the variance in BMIz was between schools. Adding age ($\hat{\beta }$ = −0.123; *SE* = 0.059) and male gender ($\hat{\beta }$ = −0.323; *SE* = 0.095) into the model explained most of the school level effect. Male gender was negatively associated with BMIz score. Adding urban or rural location of each school did not significantly predict BMIz and only explained a small amount of the school level effect (results not shown).

**Table 3:** Parameter estimates (±SE) from multilevel models predicting BMIz among Grenada School Nutrition Study students nested within 23 schools, (*N* = 689).

	**Model 1**	**Model 2**	**Model 3**	**Model 4**
**INDIVIDUAL LEVEL FIXED EFFECT**				
Age, years		−0.123 (0.059)^*^	−0.106 (0.064)	−0.116 (0.064)
Afro Caribbean		−0.001 (0.122)	0.005 (0.127)	0.005 (0.127)
Male		−0.323 (0.095)^**^	−0.264 (0.103)^*^	−1.361 (0.432)^**^
TPFI^a^			−0.175 (0.087)^*^	−0.408 (0.124)^**^
Male*TPFI				0.447 (0.171)^**^
**RANDOM EFFECTS**				
School level estimate	0.051	0.021	0.029	0.034
Individual level estimate	1.324	1.313	1.345	1.33
**FIT STATISTICS**				
AIC	2172.2	2164.9	1993.5	1988.4

a *TPFI, traditionally prepared food index*.

* *p-value < 0.05*,

** *p-value < 0.01*.

### Diet quality and BMIz

The various diet quality indices (TPFI, UPFI, and SSBI) were included in the models with age, gender, and Afro-Caribbean race. The TPFI was the only diet quality measure significantly associated with BMIz score; an inverse relationship between TPFI and BMIz score was identified ($\hat{\beta }$ = −0.175; *SE* = 0.087) (Table 3). Furthermore, a strong positive interaction was noted between gender and TPFI ($\hat{\beta }$ = 0.447; *SE* = 0.171) (Table 3). Stratified analyses were conducted to characterize this interaction. In the stratified analyses, the negative effect on BMIz score associated with eating a traditional diet was only evident in the female students ($\hat{\beta }$ = −0.395; *SE* = 0.123) (Table 4).

**Table 4:** Parameter estimates (± SE) from multilevel model 4 predicting BMIz among Grenada School Nutrition Study students stratified by gender nested within 23 schools, (*N* = 689).

	**Male**	**Female**
**INDIVIDUAL LEVEL FIXED EFFECT**		
Age, years	−0.137 (0.080)	−0.086 (0.102)
Afro Caribbean	−0.104 (0.197)	0.058 (0.162)
TPFI^a^	0.048 (0.121)	−0.395 (0.123)^**^
**RANDOM EFFECTS**		
School level estimate	0.023	0.017
Individual level estimate	1.365	1.329
**FIT STATISTICS**		
AIC	946.8	1,050.6

a *TPFI, traditionally prepared food index*.

** *p-value < 0.01*.

## Discussion

The results of this study document a low prevalence of OW/OB (17.6%) among Grenadian adolescents relative to US counterparts (27). Grenadian females demonstrated a prevalence of OW/OB half that of American girls, while Grenadian males showed a prevalence of OW/OB more than 3 times less than that of American boys. Furthermore, the mean BMIz for the study sample (mean = −0.097; *SE* = 0.045) was significantly lower than zero, indicating that on average, Grenadian adolescents had lower BMI than US adolescents in the 1980s, which is the reference population for calculating BMIz scores (27).

An additional finding in the current study suggests that diet quality is a possible etiologic factor in the epidemiologic transition from communicable disease and nutritional deficiency as the predominant causes of morbidity and mortality to a predominance of noncommunicable diseases (13, 28). Results showed an inverse association between the Grenadian TPFI and BMIz score among females in the study cohort but not in males. The TPFI assessed the number of times per week the adolescents consumed any of the common traditional foods typically prepared at home. The more times they indicated consumption of traditional foods in the past week, the lower the BMIz score. This relationship, which was not seen among males, may be attributed to the low prevalence of OW/OB among the males.

The lack of a significant effect of school location (urban/rural designation) on BMIz may be due to a few reasons. One possibility is the small sample size of urban schools, with only two schools out of the 23 schools classified as urban. In contrast, about one-third of the Grenadian population resides in an urban area (29). Another option is the collective influence of many single-sex schools within the survey cohort and the greater risk of OW/OB among females, thereby attenuating the school level effect after adding sex to the model.

These findings among adolescents are unexpected given the prevalence of OW/OB among Grenadian adults. Bansilal et al. (22) found that adult Grenadian women had a higher prevalence of OW/OB compared with US women in the NHANES (68.1 vs. 58.4%) after standardizing to the Grenadian adult population. Findings indicate that Grenadian adults have transitioned to a more OW/OB population in the absence of a high prevalence of OW/OB among Grenadian adolescents, which is multiple times lower as compared to their US counterparts (27). There is evidence that the infrastructure that permitted the introduction of ultra-processed, sugar-sweetened beverages to Grenada did not arise until the early 2000s (30), therefore the obesogenic effects may not have yet reached adolescents. Popkin et al. (31) found this is a global phenomenon, in that rates of increases in overweight were higher among adults than children in seven countries comprising high-, middle-, and low-income strata. This lag in overweight trends between children and adults may explain why Grenadian adolescents did not have a high OW/OB similar to adults. The absence of a period effect in this cohort is a possible explanation for this observation, suggesting a generational effect may be operating (32).

In populations most influenced by the global obesity pandemic, period and age effects are well-documented. However, it has also been proposed that cohort or generational effects may be operating to amplify the risk of non-communicable diseases (NCDs) for populations in affected countries ill prepared to address the pandemic (3). Hypotheses associated with a potential generational effect involve the developmental origins perspective (e.g., fetal overnutrition) (33, 34). The developmental origins perspective proposes that exposures during critical periods of development (e.g., prenatal period), when there is a high degree of genomic plasticity, can result in permanent physiologic changes predisposing the affected individual to obesity during early life (35, 36). Consequently, a period effect resulting in high rates of obesity among women of childbearing age could theoretically result in a generational effect evidenced as adolescent obesity among their offspring (32). Specifically, women who are either obese or have gestational diabetes during pregnancy provide an *in utero* environment associated with developmental overnutrition, a condition known to negatively affect a number of physiologic mechanisms (33, 37–40). Phenotypically, affected individuals have increased odds of being obese and may experience an accelerated growth trajectory during childhood due to an altered physiology (32, 40, 41). It is important to note that unlike period effects, which impact all age groups similarly and gradually occur over many years, a generational effect of this type emerges only in the cohort born to the generation of women of childbearing age who have gradually become obese (42). Consequently, the effect should be temporally lagged compared to ongoing period effects. Unfortunately, few studies have followed successive birth cohorts to document the possibility of a generational effect, much less a lag in the initiation of such an occurrence, and the methods previously employed have been contested (32, 43, 44). A better understanding of how this intergenerational amplification of risk is operating is critical to understanding the physiologic processes and societal exposures that drive the global obesity pandemic (3, 33, 37). Such information is necessary for the design of prevention strategies worldwide since it would shift the target to women of child-bearing age rather than the population as a whole.

This cohort of Grenadian adolescents assessed in 2013–2014 may have been unexposed to fetal overnutrition, the postulated origin of the generational effect, which is shown to promote childhood onset of OW/OB (32, 45). Due to the late introduction of processed food and beverages to Grenada, the mothers of this birth cohort may have escaped exposure to this type of food, along with obesity, before or during pregnancy. Thus, offspring may be protected from obesogenic promoters due to a lack of fetal overnutrition. This may explain why, unlike OW/OB in adults, a higher prevalence of OW/OB among Grenadian adolescents as compared to a US cohort was not observed. It is expected this generational effect will reverse in future generations, switching from protective to obesogenic promotion as obesity trends increase.

There are several limitations to the study. The first being its cross-sectional design, which does not allow for a determination of the temporality of OW/OB trends among the study population. Also, there is a lack of a similar comparison cohort for these Grenadian adolescents. Diet quality and adolescent OW/OB in LMIC has not been thoroughly researched, therefore we are unable to determine if our results are typical of this population in similar settings. Lastly, census data regarding Grenada is not readily available, consequently standardizing current NHANES 2013–2014 data to the Grenadian population would not be possible.

The study identified lower prevalence of OW/OB in Grenadian adolescents compared with both their US counterparts and Grenadian adults. In addition, despite low prevalence of OW/OB, adolescent Grenadian females had twice the overweight risk compared with males. Attending a rural or urban school did not have an effect on student OW/OB. Finally, adherence to a diet of traditional prepared foods was associated with lower BMIz scores among the females. However, further research in subsequent birth cohorts is needed determine if adolescent obesity will increase due to a generational effect.

## Author's note

In memory of Romero Benjamin.

## Author contributions

RS made substantial contributions to the conception, design, and analysis of the data, as well as drafting the article and approved the final version. He agrees to be accountable for all aspects of the work with regard to accuracy and integrity. RR and TN made substantial contributions to the conception, design, and data acquisition, as well as revising the article for intellectual content. He approved the final version. He agrees to be accountable for all aspects of the work with regard to accuracy and integrity. AG and TF made substantial contributions to revising the article for intellectual content. She approved the final version. She agrees to be accountable for all aspects of the work with regard to accuracy and integrity. CL made substantial contributions to the data analysis, as well as revising the article for intellectual content. She approved the final version. She agrees to be accountable for all aspects of the work with regard to accuracy and integrity. RA, NA, CR, RF, JB, JJ, and RB made substantial contributions to data acquisition, as well as revising the article for intellectual content. They each approved the final version. They agree to be accountable for all aspects of the work with regard to accuracy and integrity. RW made substantial contributions to the conception, design, and data analysis, as well as revising the article for intellectual content. He approved the final version. He agrees to be accountable for all aspects of the work with regard to accuracy and integrity. MS was the senior researcher on the project. She made substantial contributions to the conception, design, and data acquisition, as well as revising the article for intellectual content. She approved the final version. She agrees to be accountable for all aspects of the work with regard to accuracy and integrity.

### Conflict of interest statement

The authors declare that the research was conducted in the absence of any commercial or financial relationships that could be construed as a potential conflict of interest. The handling editor and reviewer JGK declared their involvement as co-editors in the Research Topic, and confirm the absence of any other collaboration.

## Abbreviations

BMI: body mass index
BMIz: standardized BMI z-score
CDC: Centers for Disease Control
GSNS: Grenada School Nutrition Study
LMIC: low- and middle-income country
NCDs: non-communicable diseases
NHANES: National Health and Nutrition Examination Survey
OW/OB: overweight and obesity
SSBI: sugar sweetened beverage index
TPFI: traditional prepared food index
UPFI: ultra processed food index.
